# Unraveling the development behind unisexual flowers in *Cylindropuntia wolfii* (Cactaceae)

**DOI:** 10.1186/s12870-022-03431-0

**Published:** 2022-03-02

**Authors:** Niveditha Ramadoss, Amy Orduño-Baez, Carlos Portillo, Scarlet Steele, Jon Rebman, Lluvia Flores-Rentería

**Affiliations:** 1 Department of Biology, San Diego State University, San Diego, USA; 2 University of Santa Cruz, San Diego, USA; 3 Department of Botany, San Diego Natural History Museum, San Diego, USA

**Keywords:** Dioecy, *Cylindropuntia wolfii*, Pollen viability, Unisexual floral development

## Abstract

**Background:**

In certain unisexual flowers, non-functional sexual organs remain vestigial and unisexuality can be overlooked leading to the ambiguous description of the sexual systems. Therefore, to accurately describe the sexual system, detailed morphological and developmental analyses along with experimental crosses must be performed. *Cylindropuntia wolfii* is a rare cactus endemic to the Sonoran Desert in southern California and northern Baja California that was described as gynodioecious by morphological analysis. The aims of our project include accurately identifying the sexual system of *C. wolfii* using histological and functional studies and characterizing the developmental mechanisms that underlie its floral development.

**Methods:**

Histological analyses were carried out on different stages of *C. wolfii* flowers and controlled crosses were performed in the field.

**Result:**

Our results identified C. *wolfii* to be functionally dioecious. The ovule and anther development differed between staminate and pistillate flowers. In vivo pollen germination tests showed that the pollen of staminate and pistillate flowers were viable and the stigma and style of both staminate and pistillate flowers were receptive. This suggests that there are no genetic or developmental barriers in the earlier stages of pollen recognition and pollen germination.

**Conclusions:**

Despite being functionally dioecious, we observed that functionally pistillate individuals produced fruits with a large number of aborted seeds. This implies that not only does this species have low reproductive success, but its small population sizes may lead to low genetic diversity.

## Background

The breeding or sexual systems of organisms is a critical aspect of natural biology that affects genetic diversity and genome evolution [1]. Dioecy is a sexual system where populations are made of distinct male and female individuals [2]. An estimated 6% of angiosperm species have evolved separate sexes in a dioecious system, and dioecy has evolved at least 871 times independently in 175 families [3, 4]. Dioecy is also reported to have evolved from hermaphroditic flowers through the accumulation of mutations that drive male sterility in pistillate flowers and female sterility in staminate flowers [3]. Despite heavily depending on pollinators for a successful fertilization [5] and having fewer individuals that can produce seeds compared to the hermaphroditic populations, dioecious angiosperms are present in exclusively species rich dioecious clades, thereby negating any negative consequences [4]. Studies on unisexual flower development have shown that the developmental processes are not consistent among species and unisexual flower development is not well studied.

In diecious species, two types of developmental regulations are involved in producing unisexual flowers [6]. Type I involves carpel/stamen abortion after all the organs have been specified by the flower, but one sexual organ remains functional (Mitchell and Diggle [6]). This leads to unisexual flowers that carry vestigial organs of the other sex [7]. Type II involves the arrest of one sexual organ at a very early stage which leads to unisexual flowers by inception [6]. Programmed Cell Death (PCD) has been proposed as a principal force for driving the development of unisexuality in angiosperms through Type I [8, 9, 10]. However, the abortion of the male and female organs can occur in different stages and tissues [11, 12, 13, 14, 15] and it is species dependent.

As abortion of one of the sexual whorls occurs late during the development of Type I flowers, non-functional sexual organs remain vestigial and unisexuality can be overlooked leading to the ambiguous description of the sexual systems. Thus, to describe the breeding system precisely, detailed morphological and developmental analyses or experimental crosses are necessary [16, 17]. For example, some species have been originally considered as gynodioecious or androdioecious, but a closer look at their hermaphroditic flowers has shown they are functionally dioecious. For example, *Spachea membranacea* Cuatrec. (Malpighiaceae) and *Withania aristata* (Aiton) Pauquy (Solanaceae) were observed to have individuals with either hermaphroditic flowers or pistillate flowers, but crossing and anatomical experiments revealed that the hermaphroditic individuals were functioning as males, thus describing their sexual system as functionally dioecious [18, 19]. A member of the Caryophyllaceae, *Honckenya peploides* (l.) Ehrh. var. *major* (Hook.) Abrams, was initially identified as androdioecious based on morphological studies and then proved to be functionally dioecious based on manual outcrosses performed over a consistent two year study period [20]. Thus, it is of importance to determine the sexual system of plants based on functional studies as their reproductive systems are often not accurately represented by superficial observations of the floral morphology [21, 18].

The family Cactaceae has about ~2000 species [22]. Most of them are hermaphroditic; although 23 species have Type I unisexual flowers in either dioecious, gynodioecious, or trioecious sexual systems ([23] and references therein). Within the cactus family, the description of the sexual systems has been ambiguous and imprecise, which is attributed mostly to anecdotal observation rather than detailed morphological analysis or experimental confirmation of the system [16]. Recent studies in the Cactaceae have advanced our understanding of the cellular mechanisms by which hermaphrodite flowers become unisexual, as well as their spatial and temporal patterns [13, 24]. In most cacti specimens, pistillate flowers underwent degradation before the completion of meiosis. For instance, in the genus *Consolea*, [25] showed that pistillate flowers underwent abortion early during the onset of meiosis leading to sterile anthers bearing no pollen grains. In *Opuntia stenopetala* Englem., the pistillate flowers undergo various cellular changes associated with PCD (vacuolization, DNA degradation, cytoplasm collapse) in the microsporangium and all cell layers prior to completion of meiosis [13]. Similarly, in four species of *Echinocereus* with pistillate flowers, the microspore mother cell development was arrested prior to meiosis with other cell layers collapsing later [24].

In Cactaceae, female sterility varies considerably between taxa [15]. In the functionally dioecious *O. stenopetala*, the ovule abortion in staminate flowers was initiated in early primordial stages [26], whereas in *Consolea*, the ovule degeneration was induced prior to anthesis and after the development of embryo sac [27]. In *Echinocereus*, the ovule abortion takes place after the zygote is formed (post-fertilization) [24]. The role of PCD in female sterility of Cactaceae is not clearly determined. One recent study in *Opuntia robusta* H.L. Wendl. ex Pfeiff. showed that placental arrest and ovule abortion in staminate flowers were regulated by PCD [15].

Plants that are dioecious can deviate from the 1:1 sex ratio, especially those restricted to small populations, thereby decreasing their viability [28, 29]. Among them, male biased ratios are at least twice as frequent as the female biased ones [28]. These biases are attributed to the differences in reproductive costs, pollen or seed dispersal, sex chromosomes and/or differential rate of mortality [28]. In Cactaceae, subdioecious populations of *Consolea spinosissima* (Mill.) Lem. [30] and *Pachycereus pringlei* (S. Watson) Britton & Rose [31] as well as functionally dioecious populations of *O. stenopetala* have been reported to possess a male biased sex ratio.

Interestingly, several species of *Cylindropuntia* have been described as gynodioecious, with some individuals having perfect flowers and others having functionally female flowers [32, 33]. These species are *C. calmalliana* (J.M. Coult.) F.M. Knuth, *C. chuckwallensis* M.A. Baker and M.A. Cloud-Hughes, *C. molesta* (Brandegee) F.M. Knuth, *C. sanfelipensis* (Rebman) Rebman, and *C. wolfii* (L. D. Benson) M.A. Baker.

**Fig. 1:**
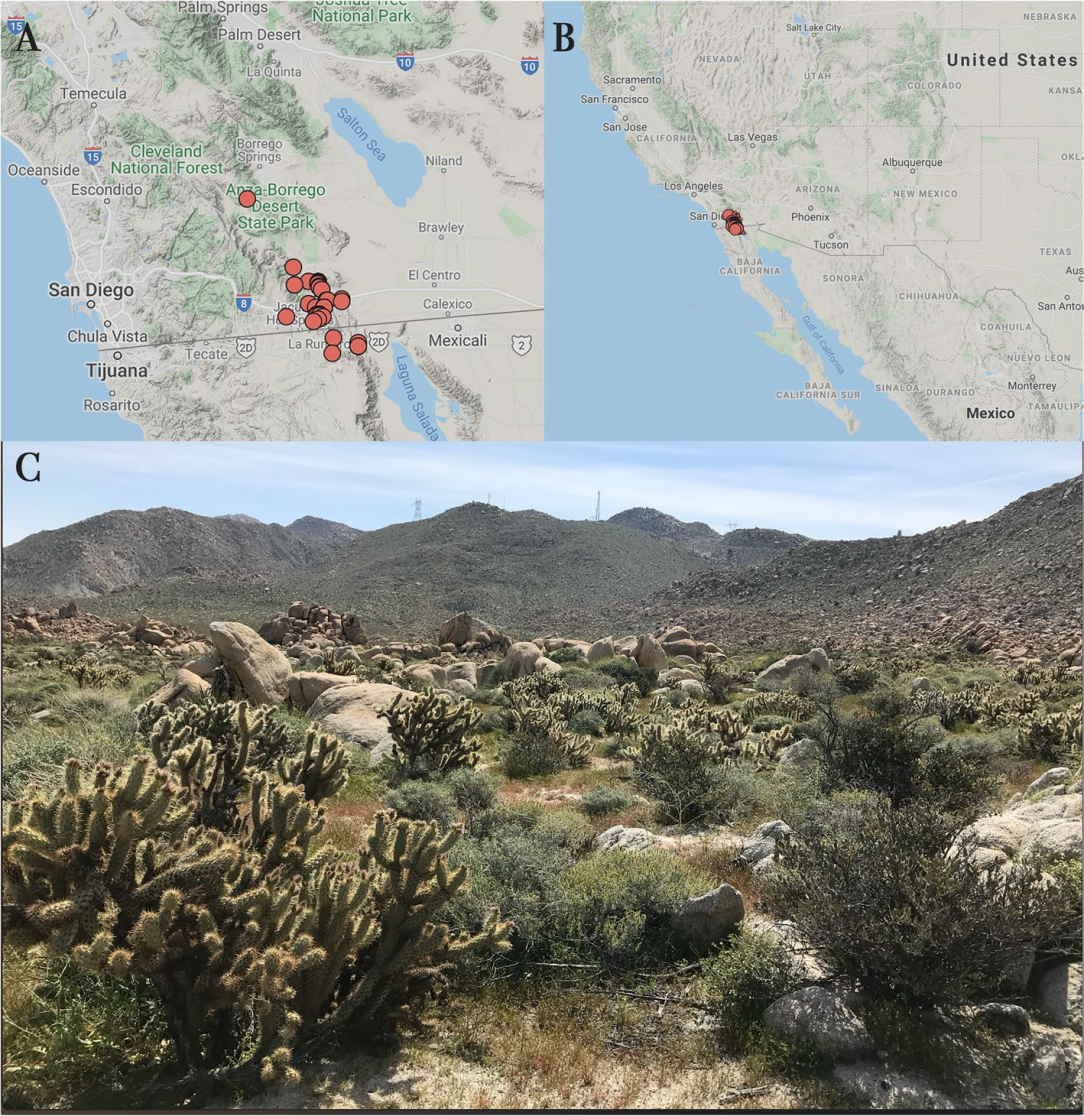
Distribution of *Cylindropuntia wolfii* (**A**). This species has a narrow distribution in both California and Baja California, Mexico (**B**). Red points show the current distribution of *C. wolfii*. Occurrence data that included only precise points were extracted from SEINet Portal (www.swbiodiversity.org), misidentifications were excluded and the map was generated using Google imagery. (**C**) Natural occurrence of *C. wolfii* in Mountain Springs, Imperial County, California. Figures A and B were obtained from SEINet Portal and were collated with figure C in Adobe Photoshop

In our study, we focus on *Cylindropuntia wolfii*, which occurs within a very restricted range in extreme southern California, USA and extreme northern Baja California, Mexico (Fig. 1). This species has been described as gynodioecious based on field and herbarium specimen observations [32], but little is known about its reproductive biology. The putative female individuals were assumed because the anthers looked shriveled and no pollen was present. Type I flowers are easily misidentified by superficial observations, therefore, histological studies and experiments (e.g., manual crosses) are needed in order to accurately describe the sexual system in *C. wolfii*. Our goals are to determine the sexual system of *C. wolfii* and to describe the developmental processes contributing to the formation of unisexual flowers. We used a variety of histological observations, experimental crosses, and pollen viability tests.

## Results

Based on histological examinations and cross-pollination experiments, we determined that *C. wolfii* is not gynodioecious as previously reported, but is instead functionally dioecious with bisexual flowers aborting one sex during the development process. Thus, the previously identified hermaphrodites are hereafter referred to as functional males or staminate flowers.

### Anther development

#### Staminate flowers

**Fig. 2:**
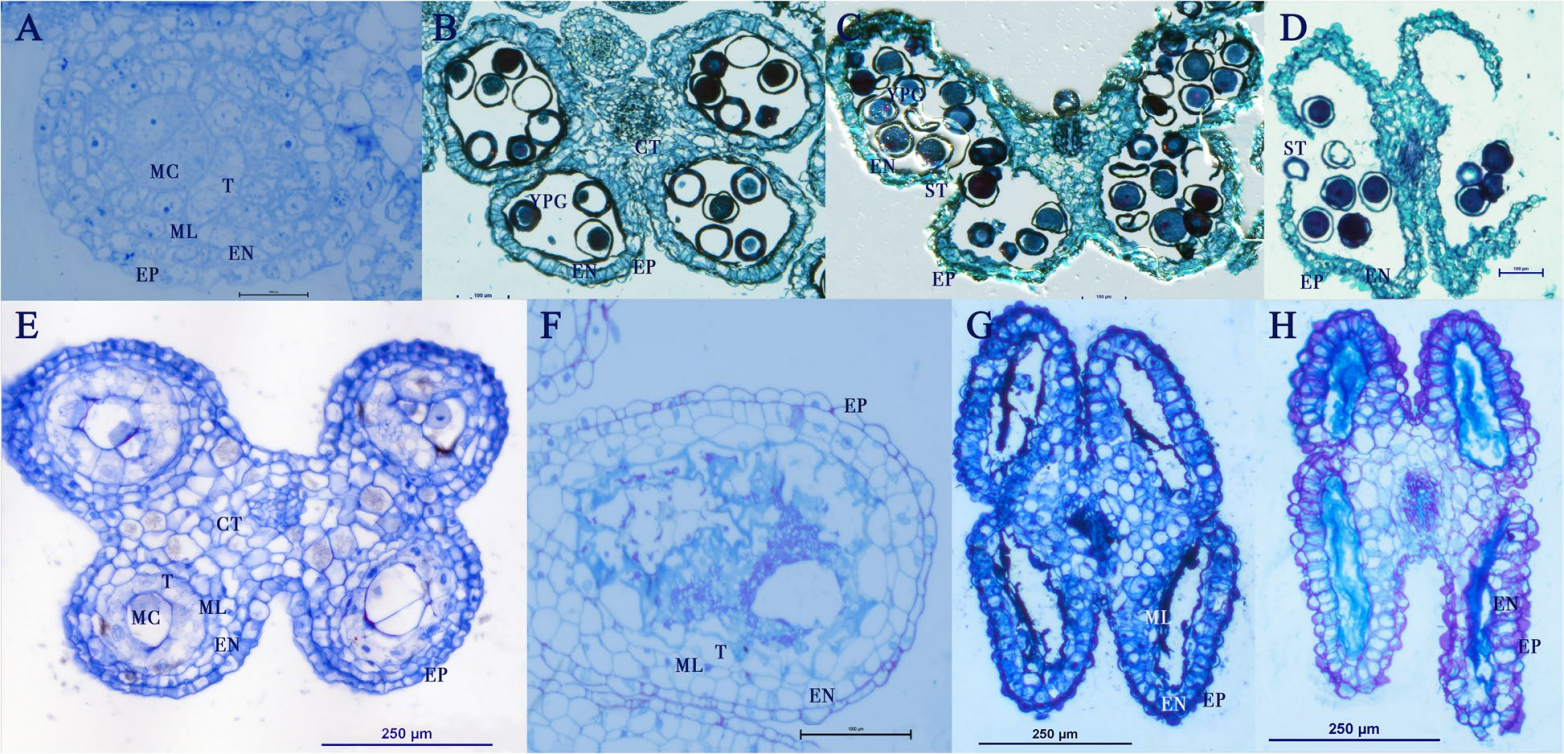
Anther development of flowers of *Cylindropuntia wolfii*. **A**) Anther of staminate flowers shows four layers at pre-meiosis of the microspore mother cells (MC). (**B**) Tetralocular male anther in pre-anthesis with young pollen grain (YPG) developing with tapetum fully disintegrated but endothecium (EN), epidermis (EP) and connective tissues (CT) intact. (**C**) Male anther in late pre-anthesis with stomium (ST) initiation indicating that the anther is about to dehisce. (**D**) Male anther at anthesis releasing fully mature pollen grains. (**E**) Female flower anther in pre-anthesis with MiMC (MC) developing. All cells in the anther wall such as tapetum (T), middle layer (ML), endothecium (EN) as well as the MiMC start showing hyper-vacuolization. (**F**) Microspore mother cells and tapetum show complete disintegration by this stage and only few tapetal cells are visible. (**G**) Female anther in late pre-anthesis showing the disintegration of the MiMC, nuclei are visible in cells of the anther wall. (**H**) Aborted anther in female flower at anthesis lacking nuclei in most remaining cells

**Fig. 3:**
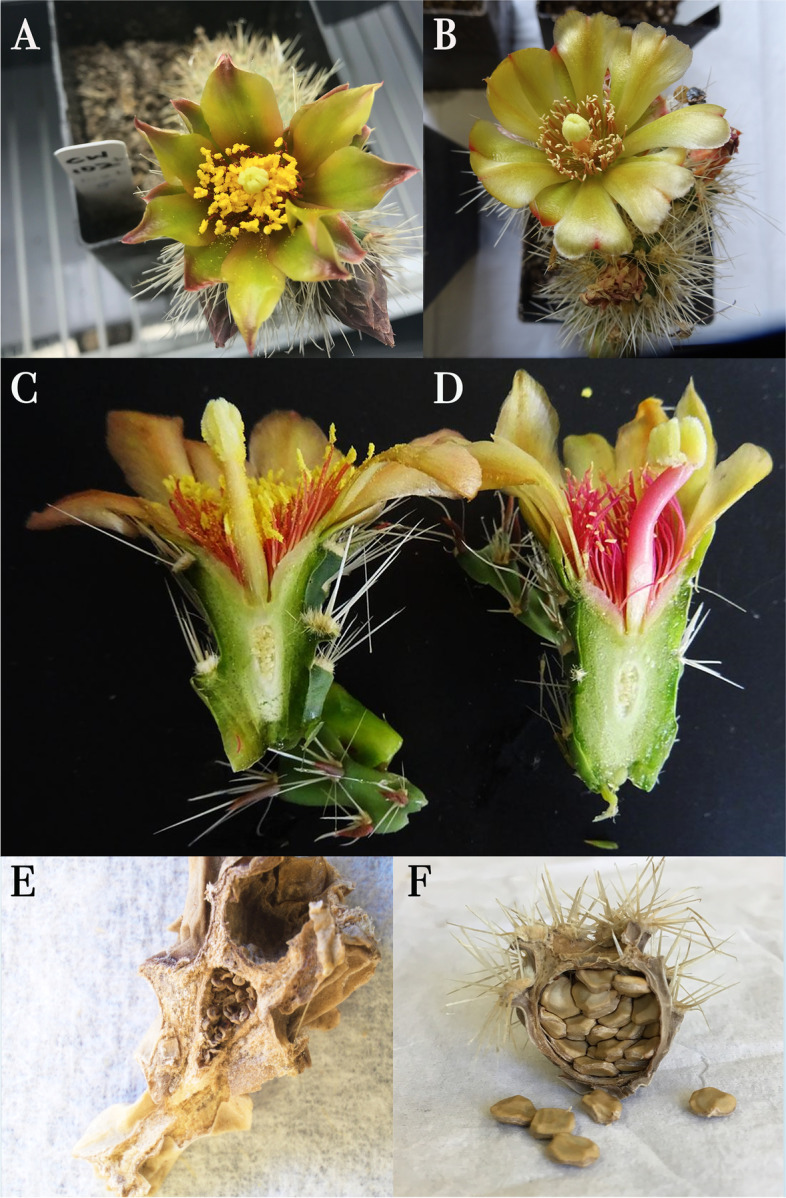
(**A**) *Cylindropuntia wolfii staminate* flower (left) and pistillate flower (right). (**B**) Lateral cross-section of staminate flower (left) and pistillate flower (right). Fruits collected from a staminate plant (**C**) and pistillate plant (**D**). Fruits collected from male (**E**) and female plants (**F**). The figures are our own, collated by Adobe Photoshop

Staminate flowers develop a typical tetralocular anther with an anther wall composed of four single cell layers surrounding the Microspore Mother Cells (MiMCs). The wall is formed by the epidermis, endothecium, middle layer and tapetum (Fig. 2 A). Meiosis of the MiMCs results in four microspores, at this point the middle layer has already disappeared and the tapetum disintegrates which contributes to the pollen nutrition and wall ornamentation of the intermediate pollen grain (Fig. 2B). The epidermal cell begins to change into a conical shape. In late pre-anthesis, the anthers become bilocular with the disintegration of the connective tissues that form the septum of the microsporangia, and a stomium forms (Fig. 2 C). During anthesis, the last layer consists of endothecium and epidermis in conical shape. The wall dehydrates, and eventually dehisces (Fig. 2D) to reveal visible pollen (Fig. 3 A and C).

#### Pistillate flowers

Anther development of pistillate flowers is interrupted after the formation of the MiMCs and does not undergo meiosis. At this stage, the MiMCs and the cells of the anther wall are highly vacuolated specially the tapetum (Fig. 2E). The MiMCs and the tapetum completely disintegrate, while the middle layer is atypically retained compared with the staminate flowers (Fig. 2 F). This is followed by the hypervacualization of the epidermis and the endothecium cells and the disintegration of their nuclei (Fig. 2 F, 2G). In pistillate flowers, the anthers at anthesis do not dehisce. The septum that separates the two microsporangia in each thecae remains visible (Fig. 2 H), whereas it disintegrates in the male counterpart (Fig. 2 C). The anther development halts, no pollen is formed, and no dehiscence occurs. Therefore, no pollen is readily visible on the anthers of pistillate flowers (Fig. 3B and D).

### Ovule development

#### Staminate flowers

**Fig. 4:**
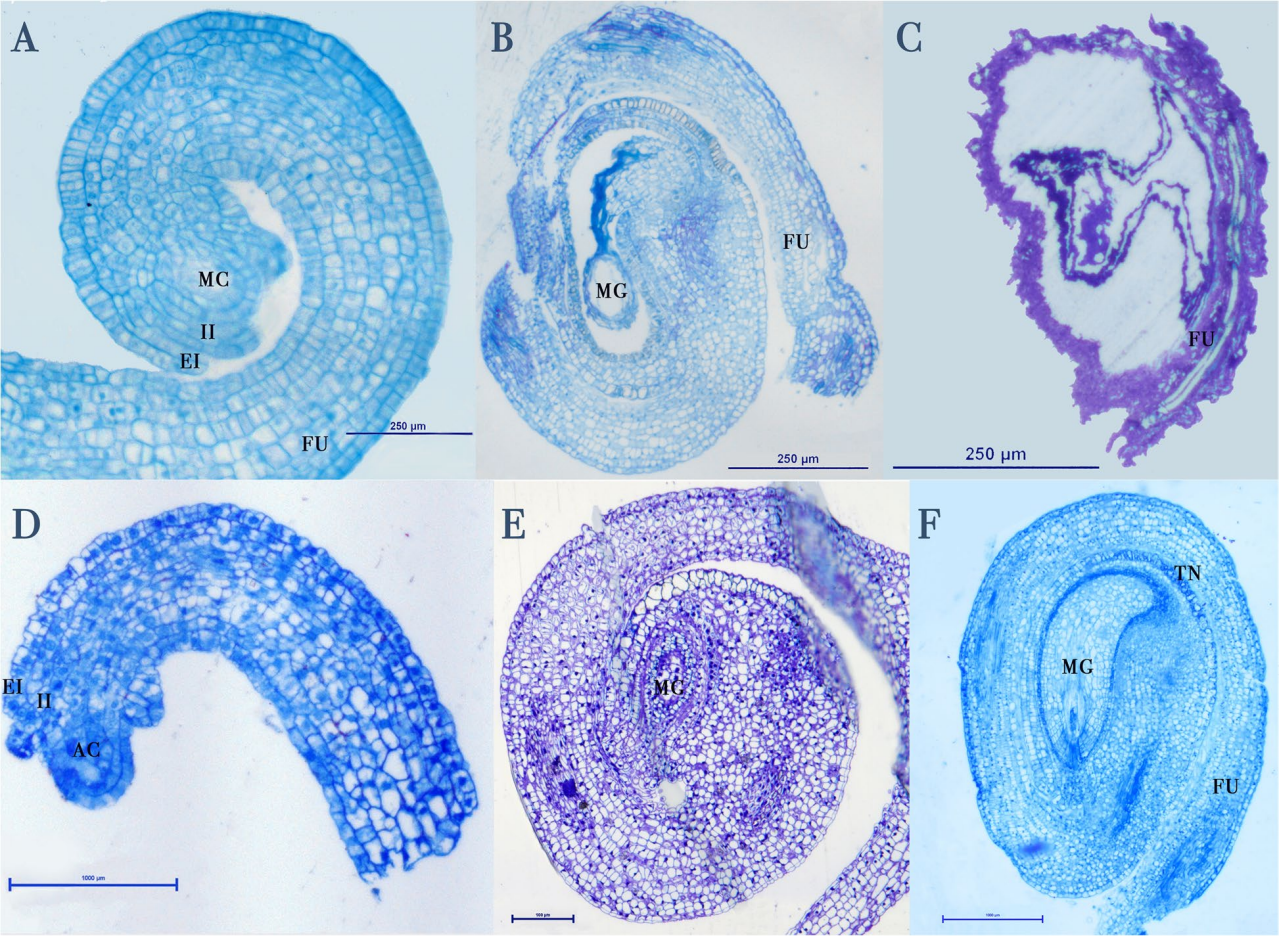
Ovule development of flowers of *Cylindropuntia wolfii*. (**A**) Ovule in male flower at pre-anthesis, with long funicle (FU) and integuments (external (EI) and internal (II)) developing with a Megaspore Mother Cell (MC). (**B**) Ovule of male flower in late pre-anthesis showing the collapse of the megagametophyte (MG). (**C**) Aborted ovule in male flower completely collapses in male flowers, at maturity only cellular debris are observed. (**D**) Ovule of female flower in early pre-anthesis, with a long funicle (FU) and integuments (external (EI) and internal (II)) developing with an archesporial cell (AC). (**E**) Ovule of female flower in early pre-anthesis showing the formation of the megagametophyte (MG). (**F**) Fully mature ovule of female flower in early pre-anthesis showing well developed megagametophyte and tannin (TN) deposits

In staminate flowers, differentiation of the ovule primordia is similar to that of the pistillate flower until the formation of the megagametophyte. The ovule primordia develop the integuments, the funicle, and Megaspore Mother Cells (MeMC) (Fig. 4 A). During the megagametogenesis, the young circinotropous ovule degenerates. The degeneration starts on the megagametophyte and extends to the nucelle (Fig. 4B). The funicle exhibited some signs of disruption but it is not known if that is a direct disintegration, or an artifact due to the lack of internal support of the ovule during the sectioning. At anthesis, the ovule is completely disintegrated, cellular debris can be observed along with tannins in the integuments and vascular system in the debris of the funicle (Fig. 4 C). Although staminate flowers’ ovaries carry ovules early in development, (Fig. 3 C) they are nonfunctional.

#### Pistillate flowers

**Fig. 5:**
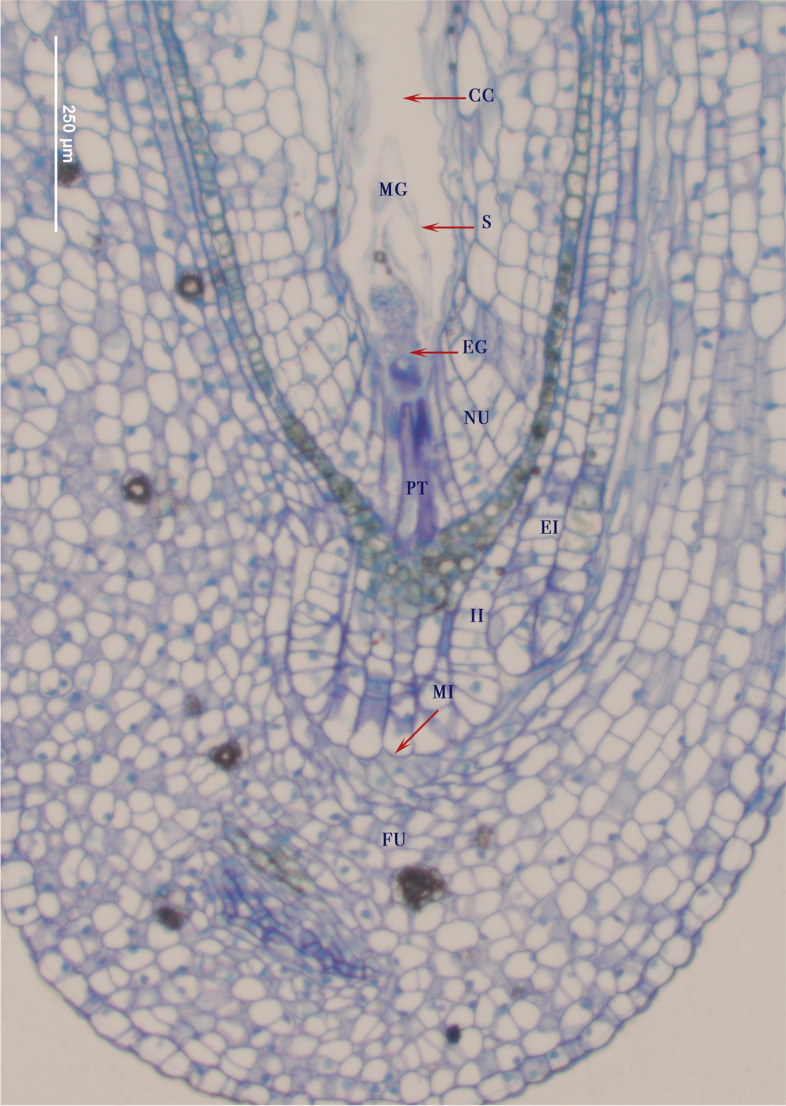
Fertilization of the egg (EG) by pollen tube (PT) in megagametophyte (MG) of ovules of female flowers. The pollen tube enters through the micropyle (MI). The megagametophyte is surrounded by nucelle (NU), internal integument (II) and external integument (EI), which in turn is surrounded by the funicle (FU). Four cells are found at maturity including the central cell (CC), two synergids (S, only one visible) and the egg (EG)

In pistillate flowers, the primordial ovule developed starting with wrapping itself around by the long funicle, forming the integuments and the MeMCs (Fig. 4D). The pre-anthesis ovule has a curved nucelle, the MeMCs divides into 7 cells to form the megagametophyte. At this stage the development of the integuments is completed with the internal integument defining the micropyle (Fig. 4E). Tannin deposits in the integument are visible during megagametogenesis (Fig. 4E). At anthesis, the fully mature ovule is campylo-circinotropous. The megagametophyte is well developed and surrounded by the nucelle. The chalazal region is formed by the conjunction of the funicle and the integuments which have tannin deposits (Fig. 4 F). The embryonic sac develops seven cells - three antipodal cells are ephemeral and only four cells are found at maturity including the central cell, synergids and the egg at the micropylar end (Fig. 5).

### Pollen-stigma interaction

**Fig. 6:**
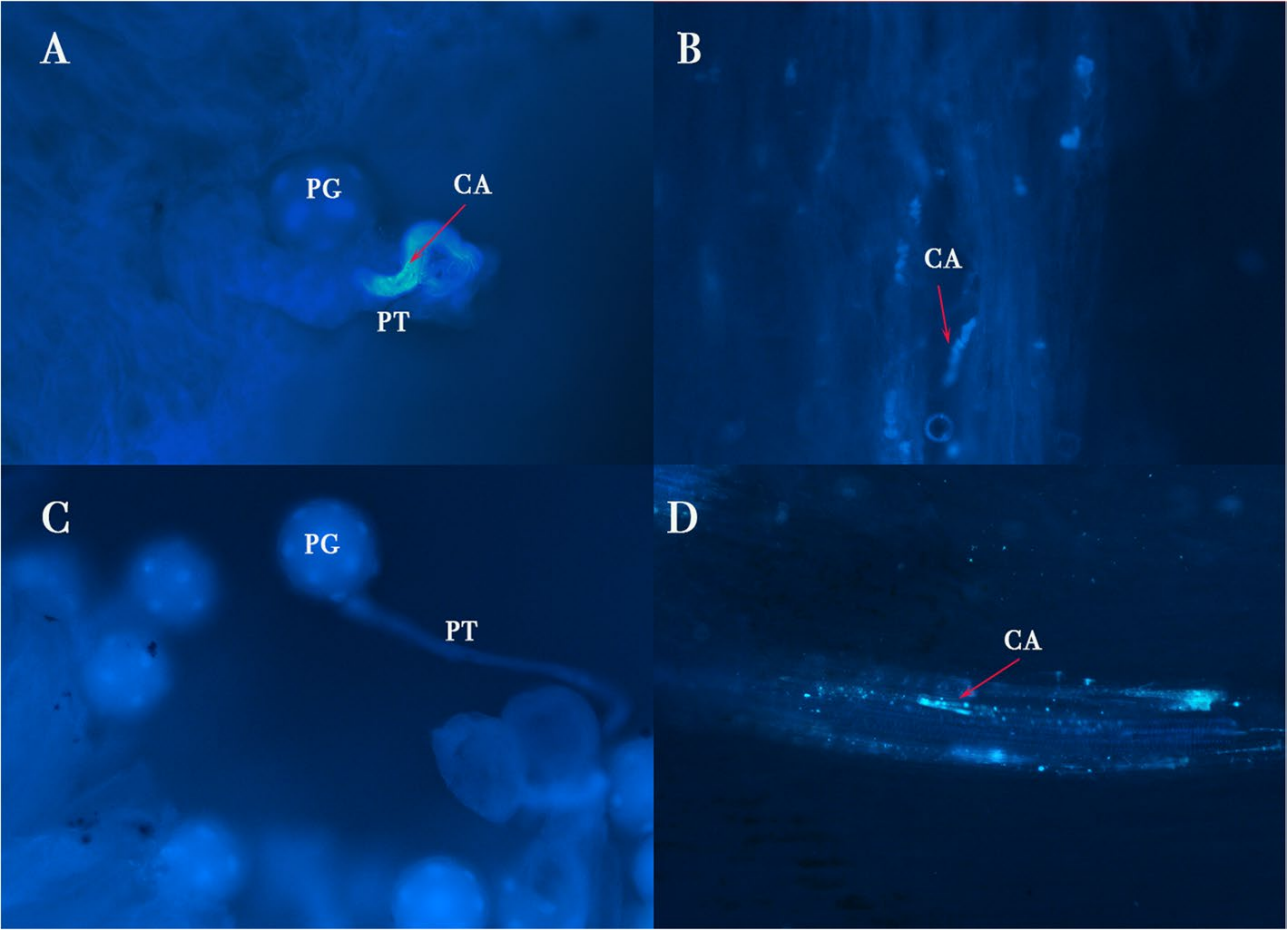
*Cylindropuntia wolfii* pollen tube (PT) development from pollen grain (PG) on stigma and style of flowers used for the crosses (**A**, **B**) male x male (selfing) male x female (**C**, **D**). The callose (CA) in the pollen tube fluoresces as a result of staining by aniline blue. Other crosses were not possible as females do not form pollen. Magnification:10X

To test the viability of the pollen and the receptivity of the stigma and style, the aniline blue dye was used to stain the callose in pollen tubes, enabling clear visualization of the germinated pollen in experimental crosses (Fig. 6). The germinated pollen tubes were observed in male × male selfed crosses (Fig. 6 A, B) and female × male crosses (Fig. 6 C, D). This germination of pollen in stigmas (Fig. 6 A, C) and style (Fig. 6B and D) of pistillate and staminate flowers indicates that the stigma and style of pistillate and staminate flowers are both functionally receptive. As pistillate flowers did not contain any pollen, there were no pollen tubes observed between male x female crosses and female x female selfed crosses.

### Fruit development and seed set from natural and artificial crosses

**Fig. 7:**
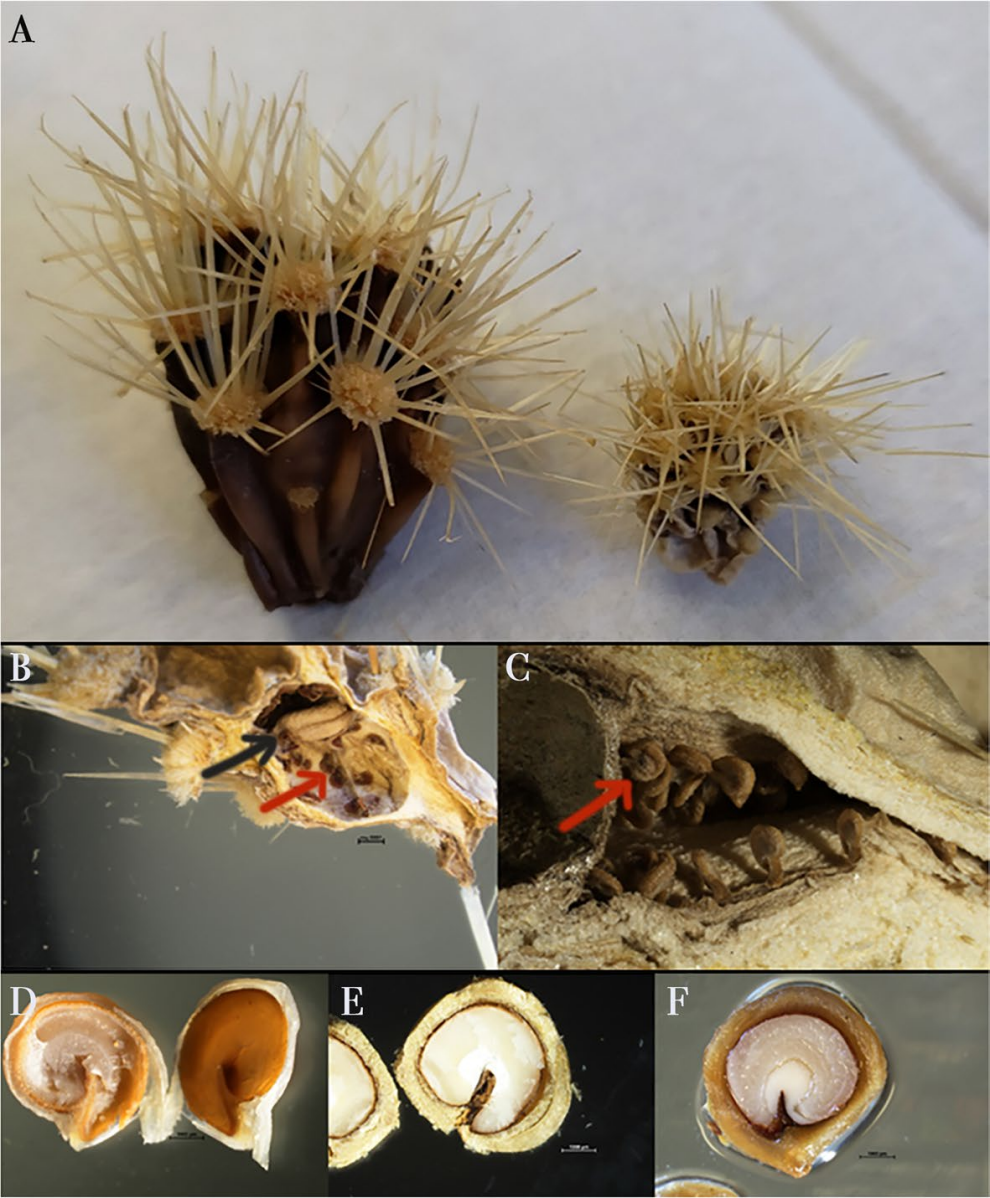
*Cylindropuntia wolfii* fruit and seed development. **A**) Fruits of female (left) and male (right) individuals. Aborted ovules are found in fruits of both female (**B**) and male (**C**) individuals (red arrows), however, fruits of female individuals also form some mature seeds (black arrow). **D**-**F**) Mature seeds from fruits of female individuals showing the thick funicular coat. **D**) Seed with abnormal development that lacks the white solid embryo as shown in E-F. The latter shows a mature seed with endosperm consumed by the fully developed embryo and remaining perisperm (arrow). The figures are our own, collated by Adobe Photoshop

To further examine which sex contributes to fruit and seed formation, field observations and experimental crosses were carried out. The fruits of *C. wolfii* become dry at maturity. Functionally female individuals were the only ones that formed mature fruits. Functionally male plants aborted the ovules, but ovaries were retained and dried (Fig. 3 C). Thus, the fruits of female individuals are larger than male aborted fruits (Fig. 7 A). However, many fruits were also aborted in functionally female plants, and some produced a few mature seeds with most ovules aborted, flattened, and located against the wall of the ovary (Fig. 7B). In contrast, no mature seeds were produced in functionally male individuals, (Fig. 7 C). Some mature seeds of functionally female individuals lacked embryos (Fig. 7D), but most developed mature embryos (Fig. 7E F). Since aborted ovules were visible under the microscope in both female and male fruits, we were able to estimate the seed set in both sexes in manual and natural crosses.

**Fig. 8:**
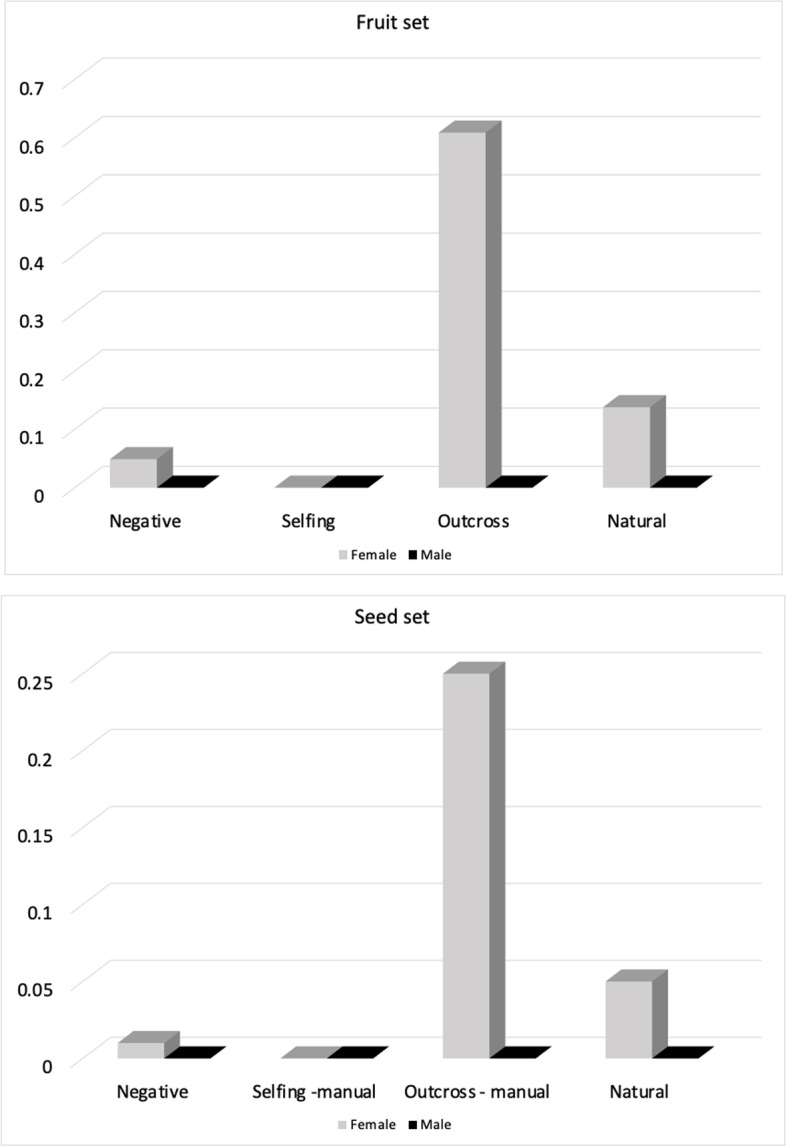
Experimental crosses in *Cylindropuntia wolfii*. (**A**) Bar chart showing the average fruit set (Y axis) obtained from various controlled crosses (X axis) in functionally female (gray bars) and functionally male (black bars) individuals. (**B**) Bar chart showing the average seed set (Y axis) obtained from various controlled crosses in functionally female (gray bars) and functionally male (black bars) individuals

Using the controlled crossing experiment, we confirmed the ovule viability by formation of seeds. Fruits were collected when they appeared dry, plump, and brown (Fig. 7 A). The fruit set of males was 0 in all types of crosses and for females the values are as follows - (1) natural controls had a fruit set of 0.14; (2) outcross - 0.61; (3) selfing - 0 and (4) negative controls had 0.05 (Fig. 8A). From the collected fruits, we calculated the average seed set for each type of cross (Fig. 8B). The seed set values of females acting as pollen receptors were as follows: (1) natural controls had a seed set of 0.05; (2) outcross had a seed set of 0.25; (3) selfing had a seed set of 0 and (4) negative controls had a seed set of 0.01. Male individuals acting as pollen receptors had a seed set of 0 in every cross type. Our data showed that the staminate flowers produced no fruits or seeds in any type of crosses which denotes that even though they superficially appear to have a functional stigma and style, their ovules were not viable. On the other hand, we observed pistillate flowers to produce fruits and seeds in outcrosses, natural crosses and also in a single negative control individual. Overall, our results suggest that the pistillate flowers have viable ovules. Both sexual morphs subjected to selfing did not produce any seeds. This further supports that the sexual system of *C. wolfii* is functionally dioecious.

Our analysis shows that in general, the seed set of *C. wolfii* is low, 0.25 for outcrosses and 0.05 for natural crosses. This difference in seed set between natural and outcrosses is significantly different (t = 3.2568, df = 50, p-value = 0.002027).

### Sex ratio

Once we determined the sexual system, we estimated the sexual ratio in this species. A total of 137 plants were surveyed in the Mountain Springs area, 42 were identified as female individuals (31%) and 95 were identified as males (69%). The sex ratio of male: female was calculated to be 42:95, therefore there are approximately 2 males for every female in the sampled population. The male biased sex ratio is significant with P < 0.0001, df=1.

## Discussion

Given the recent evolution and wide polymorphism in sexual systems, Cactaceae is considered an ideal family to study sexual systems in plants [15]. Using histological observations, pollen viability tests, and controlled experimental crosses, we identified the sexual system of the rare cactus, *Cylindropuntia wolfii*, as functionally dioecious and not gynodioecious (individuals with hermaphroditic flowers and individuals with pistillate flowers) as previously described [32]. This species has a Type I flower, with functionally female individuals retaining aborted anthers and functionally males retaining aborted ovules. In the staminate flowers of *C. wolfii* the differentiation of the ovule primordia is similar to that of the pistillate flower until the formation of the megagametophyte. During megagametogenesis the young circinotropous ovule degenerates starting on the megagametophyte and extending to the nucelle. At anthesis, the ovule is completely aborted, but it is retained. The retention of the ovules on staminate flowers during anthesis, makes it difficult to identify whether they are aborted, reinforcing the idea that histological analyses are needed to accurately characterize the sexual system. The female sterility pattern exhibited in *Cylindropuntia wolfii* is similar to the one presented in *Consolea spinosissima* in which the ovules abort just before anthesis and start with the disintegration of nucelle proceeding to a complete breakdown of the ovule (Strittmatter et al. [30]). In comparison, in both *O. robusta* and *O. stenopetala* female sterility happens much earlier suppressing the ovule in its primordial stage [26, [15]. On the other hand, in *Echinocereus* the ovule abortion takes place only after the zygote is formed (post-fertlization) [24], requiring careful observation to determine the sexual system. These observations imply that the cellular mechanisms or pathways behind ovule abortion in *C. wolfii* might be different from that of other species in the Cactaceae.

From our histological data, it was revealed that the androecium development in pistillate flowers was interrupted in pre-anthesis with complete degradation of the tapetum and MiMCs by late pre-anthesis. The early degradation of the tapetum is known to affect the nutrient supply to developing microspores by causing variations in callose metabolism and other tapetal enzymes critical for normal development [34, 35, 36]. This pattern of tapetal disintegration is common in dioecious species of Cactaceae studied thus far although the temporal patterns might differ [15]. The mechanism behind tapetal disintegration in most of the cacti species - *Opuntia robusta* [15], *O. stenopetala* [13], and in the dioecious species of *Echinocereus* [24] is reported to be PCD. Moreover, tapetal disintegration by itself is a hallmark of PCD because mutations in male developmental genes lead to premature PCD or retarded PCD ultimately damaging the tapetum [22]. In conclusion, tapetal disintegration to attain male sterility might be a conserved trait of the Cactaceae.

Similar to *C. wolfii, O. robusta* and six species of *Consolea* have staminate flowers that appear to be hermaphroditic having a functional looking stigma and style. Our pollen germination tests showed that the stigma of staminate flowers in *C. wolfii* is functional which is also true for *O. robusta* and *Consolea* [37, 27, 15]. This suggests that there are no genetic or developmental barriers in the earlier stages of pollen recognition or pollen germination in *C. wolfii.* In contrast, the staminate flowers of *O. stenopetala* are devoid of stigmatic tissues suggesting a strict self-incompatibility mechanism [26]. Thus, the degree of degradation in stigma and style differs among cactus species. The ovule abortion is the main cause for female sterility in most species of Cactaceae although the timing and degree of abortion differs. Although in *O. robusta*, female sterility was regulated by PCD [15], it is unclear if this mechanism is conserved throughout the Cactaceae.

The controlled experimental crosses showed that functional males were not capable of producing seeds through any crosses, further confirming that their ovules are non-functional. The pistillate flowers produced seeds in outcrosses, natural crosses, and also in one functional female used as a negative control (bagged flowers). The seeds found in the female negative control could result from an improperly bagged flower that allowed insect visitation or a sign of apomixis (i.e., adventive embryony) - which is the formation of seeds without pollination and this kind of reproduction has been reported previously in a variety of *Opuntia* species [38] and in *C. fulgida* (Engelm.) F.M.Knuth [39]. Overall, our results suggest that the pistillate flowers have viable ovules but staminate flowers abort the ovules and are unable to produce seeds. This confirms that the sexual system of *C. wolfii* is functionally dioecious.

The seed set data from controlled crosses showed that, in general, the seed set of *C. wolfii* is low, and has been reported to produce generally abortive seeds [40], but very little is known about its reproductive biology. A similarly low value of seed set (avg: 37 seeds/ 158 ovules = 0.23 seed set) has been observed in *O. microdasys* (Lehm.) Pfeiff. where sexual recruitment by seeds is rare. Moreover, the seed set of *C. wolfii* was significantly lower in natural crosses as compared to manual crosses. This suggests that, in 2019, natural pollination may not be as effective as manual pollination in *C. wolfii*, possibly because of a low number of pollinators. Ecological studies are needed to ascertain the factors causing the reduced seed set in our system. About 40% of our outcrossed fruits failed to produce seeds. Some species of Cactaceae are narrowly distributed and dioecy could evolve to avoid the negative effects of inbreeding depression [41]. If dioecy prevents inbreeding depression through avoidance of selfing then why is the seed set of *C. wolfii* low? The low seed set suggests *C. wolfii* is struggling for sexual reproduction and the lack of easily detached segments suggests it does not use clonal propagation as its main way of reproduction. These combined might explain its restricted distribution. This, in turn, might lead to low levels of genetic diversity and high levels of inbreeding [42, 43] reducing the adaptive potential, and population genetic studies are necessary to address this concern and develop conservation strategies.

The sex ratio of the *Cylindropuntia wolfii* population in Mountain Springs (Imperial County) was significantly male-biased. Among dioecious species, a male-biased sex ratio is twice as common as the female-biased one [28]. A high female reproductive investment may be the cause of the male-biased sex ratio [44]. In order to precisely identify the causes of a male-biased ratio in *C. wolfii*, we need to evaluate the stage of lifecycle in which the bias is established and factors that contribute to sex ratio differences such as life history traits (survival, growth, flowering, clonality, etc.) and phylogenetic relationships [28]. One of the consequences of a skewed sex ratio is a lower effective population size that can lead to bottleneck effects. Theory predicts that male biased populations have a lower inbreeding than the female biased populations [45]. Experimental studies would be of interest to test this hypothesis and ascertain if that is why most dioecious plants are male biased.

The evolution of sexual separation and its association to polyploidy has been known for a long time [46], but it is uncertain whether it is a cause, a consequence or both. This relation is also seen in many species of Cactaceae. For example, *Pachycereus pringlei*, *O. robusta, Consolea* species and five species of *Echinocereus* have a sexual system with unisexual flowers (e.g., trioecious, gynodioecious or dioecious) and have a level of polyploidy either as tetraploid, hexaploid or octaploid [47, 48, 49, 50, 51]. This pattern is consistent in the genus *Cylindropuntia* in which polyploid species are either gynodioecious, e.g., *C. chuckwallensis, C. sanfelipensis, C. calmalliana*, and *C. molesta* (see [52]) or functionally dioecious such as in *C. wolfii*. Exceptions to this rule include the subdioecious and diploid *O. stenopetala* and *O. grandis* Pfeiff. [52], the apparently hermaphroditic and the triploid *C. arbuscula* (Griffiths) F.M.Knuth, which seems to propagate mostly clonally [52, 53], other hermaphroditic species such as *C. bigelovii* (Engelmann) F.M.Knuth is mostly triploid and rarely diploid, *C. prolifera* (Engelmann) F.M.Knuth is triploid and *C. cholla* (F.A.C.Weber) F.M.Knuth is mostly diploid and rarely tetraploid [54]. However, *C. wolfii* was previously described as gynodioecious but in this study we have shown that it has a functionally dioecious system. Therefore, a detailed study is warranted for the other polyploid species of *Cylindropuntia* whose sexual systems are described based on superficial observations *(e.g., C. arbuscula, C. calmalliana, C. chuckwallensis, C. molesta, C. sanfelipensis).*

## Conclusions

Our study is the first investigating the developmental mechanism of unisexuality in the genus *Cylindropuntia*. Through controlled cross experiments, histological analysis, and pollen viability tests, we identified the sexual system of *C. wolfii*, a rare and endemic cactus of the Sonoran Desert, as functionally dioecious rather than gynodioecious as previously described. Furthermore, we described the developmental process of unisexual flowers showing that the ovule abortion in staminate flowers and pollen abortion in pistillate flowers were asynchronous, occurring during and before anthesis respectively. Interestingly, the stigma and style of the functional staminate flowers remains viable suggesting this species controls female abortion at the ovule level only. Even though the pistillate flowers are functional there was a low seed set in both natural and manual crosses of *C. wolfii* suggesting that this species might be struggling to reproduce sexually which might explain its restricted distribution. The reduced sexual reproduction of this species and its small distribution might result in low genetic diversity and it should be further investigated. Field and genetic studies will offer insights into the consequences of dioecy in this species.

## Methods

### Plant Material

*Cylindropuntia wolfii* is listed on the Inventory of Rare and Endangered Plants by the California Native Plant Society, rare plant program [55] at CA Rare Plant Rank 4.3 due to its limited distribution, but the populations are not very threatened. It is narrowly distributed in the Sonoran Desert of southern California and Baja California [56] (Fig. 1). It is a hexaploid succulent shrub that is densely branched with stout cylindrical stems having terminal branchlets that are not easily detached. The stems have dense spination and oblong to obovate tubercles bearing the spines [57]. The flowers have a variable tepal color on different individuals ranging from yellow-bronze to red with a pink to red style and stamens with yellow to red filaments. The fruits are dry at maturity, densely spined to bur-like, and most often do not contain seeds.

Dr. Rebman undertook the formal identification of *C. wolfii* samples. We have permission to collect samples from the Schultz family at the privately owned Desert View Tower (Imperial County, CA) (32.6592 N, 116.0999 W). A permit from the Bureau of Land Management (CAD07-F-2018.00000001) issued to Dr. Jon Rebman for scientific and educational purposes was used to collect material at Mountain Springs (Imperial County, CA) (32.674509 N, 116.098834 W). We tagged 47 individuals, 27 putative hermaphrodites (flowers with developed anthers which released pollen at anthesis and a normal pistil, Fig. 3 A, C) and 20 morphologically female (with reduced anthers and a lack of pollen at anthesis, Fig. 3B and D). The flowers were collected at different development stages, ranging from early pre-anthesis to late post-anthesis based on the presumed sex of the parent plants via observed flower morphology. A voucher specimen has been deposited in the San Diego State University Herbarium (CCH1 specimen number SDSU22474).

### Histological methods

 To analyze the morphological development, flowers and buds from *C. wolfii* were collected at different stages for separating and sectioning the anthers and ovules. Some flowers were placed in plastic vials containing formalin-acetic acid‐alcohol (FAA). These samples for sectioning were dehydrated in an ascending series of ethanol and transferred to xylene substitute and embedded in paraffin. Blocks were sectioned at 10 μm and stained with safranin and fast‐green [58]. The samples were fixed in 4% paraformaldehyde in 1× phosphate-buffered saline (PBS). This was followed by dehydration using ethanol in increasing concentrations. Fixed samples were embedded through LR White Resin medium grade (Electron Microscopy Sciences, Fort Washington, PA, USA). LR-White-embedded samples were sectioned at 1-3 μm using an ultramicrotome (Reichert- Jung Ultracut E) and stained with toluidine blue solution (1% toluidine blue and 1% sodium borate in distilled water) using [59] procedure in Polychrome Stains for High Resolution Light Microscopy. Stained samples were mounted with Kleermount® solution. The mounted samples were viewed under a compound microscope (Nikon Microphot-FX) at different objectives.

### In-vivo pollen germination test

In order to test for pollen viability and stigma receptivity, a pollen germination test was performed. Manual crossing was done between different sexes at the time when the flowers were fully open. The flowers were collected approximately 48 h after the manual crossing and the pistils were removed. The pistils were prepared for analysis by following the procedure mentioned by [60] with a few modifications. The pistils were fixated in 3:1 ethanol: acetic acid solution for 24 h. After fixation, the samples were cleared by immersion in 1 M sodium sulphite for 25 min. This was followed by immersion in 1% aniline blue overnight. The pistils were then sectioned longitudinally and flattened on a clean glass slide. Glycerol at 50% concentration was used as the mounting medium. The samples were viewed with a compound microscope (Nikon Microphot-FX) at 10X objective under UV light.

### Crosses

To further test for ovule and pollen viability, controlled crosses were conducted between the putative hermaphroditic and female individuals in the field. A total of 130 crosses were performed. Plants were surveyed in early March 2018 and 2019 for floral buds, and in April 2018 and 2019 pollination tests were performed. Crosses were divided into 4 types: (1) natural controls – open pollinated flowers, (2) outcross – pollen from hermaphroditic flowers was manually deposited on stigmata of pistillate flowers and anthers from pistillate flowers were rubbed on the stigmas of hermaphroditic flowers as pistillate flowers lack pollen, (3) selfing - manually pollinated self-crosses for hermaphroditic and pistillate flowers and (4) negative control – bagged flowers to prevent natural and artificial pollination. Each cross was noted with a colored tag and protected with fabric bags (ULINE, 2018). A monthly survey was conducted, and dry fruits were collected from August-September 2018 and August-September 2019. Ovule counts, developed seed counts, and terminated ovule counts were done using a Nikon SMZ25 stereoscopic microscope. The seed set was calculated as the ratio of developed seeds with respect to the total number of ovules. A value of 0 indicates that no seeds were formed or no ovules were fertilized and a value of 1 indicates that all the ovules were fertilized to form seeds. A Welch two sample t-test was performed in the R program [61] to check if the seed set mean of outcrosses vs. natural crosses differed significantly between the pistillate flowers. The fruit set was calculated as the ratio of fruits with seeds with respect to the total number of flowers. A value of 0 indicates that no fruits were formed and a value of 1 indicates that all the flowers developed to fruits.

### Sex Ratio

We surveyed the plants present in a 600 m transect in Mountain Springs, Imperial County, California. The sexes were identified through the presence/absence of dehiscent anthers releasing mature pollen at anthesis and in some flowers through the presence of ovules in flowers and confirmed by performing viability tests and cross sections in the lab as described above. The observed sex ratio was compared to the expected 1:1 sex ratio through chi square test in MS Excel using CHISQ.TEST function.

## Data Availability

Data sharing not applicable to this article as no datasets were generated or analyzed during the current study.

## Abbreviations

PCD: Programmed Cell Death
MiMC: Microspore mother cell
MeMC: Megaspore mother cell
